# A Fluorescence Based-Proliferation Assay for the Identification of Replicating Bacteria Within Host Cells

**DOI:** 10.3389/fmicb.2018.03084

**Published:** 2018-12-12

**Authors:** Ronald S. Flannagan, David E. Heinrichs

**Affiliations:** Department of Microbiology and Immunology, University of Western Ontario, London, ON, Canada

**Keywords:** phagocytosis, *Staphylococcus*, phagolysosome, fluorescence, microscopy, macrophage

## Abstract

Understanding host pathogen interactions is paramount to the development of novel antimicrobials. An important facet of this pursuit is the accurate characterization of pathogen replication within infected host cells. Here we describe the use of a fluorescence-based proliferation assay to identify intracellular populations of replicating bacteria at the subcellular level. Using *Staphylococcus aureus* as a model Gram-positive bacterial pathogen and macrophages as a model host phagocyte, we demonstrate this assay can be used to reliably identify individual phagocytes that contain replicating bacteria. Furthermore, we demonstrate this assay is compatible with additional cellular probes that enable characterization of cellular compartments in which replicating bacteria reside. Finally, we demonstrate that this assay facilitates the investigation of both Gram-negative and Gram-positive bacteria within host cells.

## Introduction

The interaction of macrophages with bacteria often leads to ingestion of the microbe through phagocytosis which culminates with the formation of a membrane-bound organelle containing the microbial prey (Underhill and Ozinsky, 2002; Fairn and Grinstein, 2012). This vacuole, otherwise known as a phagosome, is not antimicrobial *per se*, however, through a complex sequence of interactions with the endo-lysosomal network, phagosomes mature into phagolysosomes that are markedly acidic and microbicidal (Flannagan et al., 2015). Not surprisingly, many successful pathogens with an intracellular lifestyle have evolved mechanisms to perturb phagolysosome formation or to resist phagolysosomal killing to promote bacterial survival (reviewed in Flannagan et al., 2009; Sarantis and Grinstein, 2012).

The molecular mechanisms employed by bacteria to promote intracellular survival can vary dramatically and, not surprisingly, this can impact on replication kinetics, where replication occurs, and host cell viability. For instance, *Listeria monocytogenes* and *Staphylococcus aureus*, which both can replicate within macrophages, do so in entirely different intracellular compartments (i.e., the cytoplasm and phagolysosome, respectively) (Portnoy et al., 1988, 2002; Flannagan et al., 2016, 2018a). In contrast, *Staphylococcus lugdunensis*, which remains viable within macrophages, fails to replicate altogether (Flannagan et al., 2018b). Due to these interactions, it is essential for investigators to have the tools to accurately establish whether phagocytosed bacteria replicate and to establish in which subcellular niche replications occurs. Routinely this entails performing phagocytosis or cell invasion assays followed by lysis and plating for colony forming units at various times post-infection (Edwards and Massey, 2011; VanCleave et al., 2017). While this approach will indicate whether a pathogen demonstrates robust growth, unique differences between individual host cells will be lost. Moreover, if the pathogen growth occurs in a subset of cells, proliferation may go unrecognized as colony counts are averaged over an entire host cell population.

**FIGURE 1:**
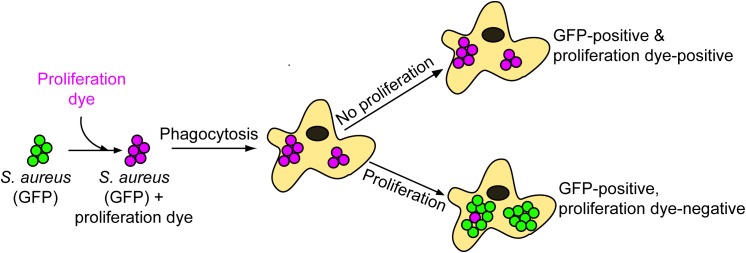
Schematic representation of the eFluor-proliferation assay. When setting up an infection GFP bacteria (or a red fluorescent protein) are incubated with amine-reactive eFluor^TM^-670 rendering the bacteria positive for both fluorophores. These double positive bacteria are incubated with macrophages and after phagocytosis and treatment with gentamicin, infected macrophages are imaged by fluorescence microscopy. The appearance of bacteria that are GFP-positive yet eFluor^TM^-670-negative indicates that bacterial replication has occurred. In contrast, bacteria that remain positive for both fluorophores have not replicated. Through replication, the proliferation dye becomes increasingly diluted between daughter cells and is eventually undetectable.

To obtain further insight into the host-pathogen interaction, fluorescence microscopy is often employed which can permit visualization of infected cells and aid in the localization of bacteria to subcellular niches. Using approaches such as this we, and others, have characterized the intracellular compartment in which phagocytosed *S. aureus* resides within macrophages (Jubrail et al., 2015; Tranchemontagne et al., 2015; Flannagan et al., 2016). However, through investigation of *S. aureus* within macrophages we uncovered difficulty in accurately distinguishing bacteria that are replicating from those that are not. Therefore, we have developed a fluorescence-based bacterial proliferation assay that allows for the accurate identification of intracellular cocci that are proliferating and present this method here (Flannagan et al., 2016, 2018a,b). This assay is based on the principle that bacteria that are covalently labeled with a finite amount of a fluorescent compound or fluorophore will remain fluorescently labeled so long as they fail to replicate (Figure 1). In contrast, bacteria of the same starting population that commence replicating will eventually appear negative for fluorescence because continued cell division results in dilution of the fluorescent compound between daughter cells (Figure 1). Importantly, this assay can be utilized in parallel with additional cellular probes such as Lysotracker^TM^ staining or LAMP-1 immunofluorescence to identify and characterize the niche in which replicating bacteria reside (Flannagan et al., 2016, 2018a,b). Here we describe this assay in detail and provide salient examples of its application to the characterization of *S. aureus* in context of the aforementioned cellular probes. Furthermore, we demonstrate that this approach can in principle also be employed for the analysis of Gram-negative bacteria. This approach should be applicable to the study of a variety of Gram-positive and Gram-negative bacterial pathogens.

## Materials and Methods

### Maintenance and Growth of *S. aureus* USA300 for Cellular Infection

*Staphylococcus aureus* strain USA300 LAC, cured of its endogenous antibiotic resistance plasmid, was maintained as a frozen stock in tryptic soy broth with 15% (v/v) glycerol. This strain was also transformed with one of a few constitutive GFP-expression vectors (described in Table 1) that have previously been described to render the entire bacterial population GFP-positive. From the frozen stock *S. aureus* USA300 was streaked onto Tryptic Soy Broth plates containing 1.5% (w/v) agar and antibiotics as appropriate and incubated at 37°C overnight (∼ 16 h) until isolated colonies were visible. This plate, kept at room temperature, was used to initiate over-night broth cultures of *S. aureus* for a period of 3 days where after that a new streak plate was created from the frozen bacterial stock. Overnight cultures of *S. aureus* were started from an isolated colony that was inoculated into a 20 mL sterile glass test tube containing 5 mL TSB with antibiotics as needed. Subsequently the culture was incubated at 37°C with continuous shaking at 250 rpm for ∼16 h yielding an overnight culture with an optical density at 600 nm (OD_600__nm_) of ∼8 to 10. For growth of *S. aureus* in the presence of antibiotics the following concentrations were used: erythromycin and lincomycin, 3 and 20 μg/mL, respectively; chloramphenicol, 12 μg/mL.

**Table 1:** Bacterial species and plasmids.

Bacterial species	Description	Source
*Staphylococcus aureus* USA300	USA300 LAC; hypervirulent community-associated MRSA; cured of antibiotic resistance plasmid.	Laboratory stock
*Yersinia pseudotuberculosis*	Wild-type, ATCC 4284	Laboratory stock
*Citrobacter rodentium* DBS100	Wild-type	Dr. B. Coombes McMaster University
*Escherichia coli* DH5α	F^-^ϕ 80d*lac*ZΔM15 *recA1* *endA1* *gyrA96* *thi-1 hsdR17* (rK^-^ mK^+^) *supE44 relA1 deoR* *Δ(lacZYA-argF*)U169 *phoA* λ	Laboratory stock
*E. coli* ML35		Dr. S. Koval, Western University
**Plasmids**		
PprsA::gfp	Constitutive *S. aureus* GFP expression vector; Amp^r^ Ery^r^	Sayedyahossein et al., 2015
pCG44	*E. coli*/*S. aureus* shuttle vector for constitutive expression of pHluorin in *S. aureus* and used here as a GFP expression vector; Amp^r^ Cm^r^	Gries et al., 2016
pFPV25.1	Constitutive GFP expression vector with the promoter of *rpsM* from *S. typhimurium* driving GFPpmut3; Amp^r^	Valdivia and Falkow, 1996


### Labeling of *S. aureus* With the Fluorescent Proliferation Dye

To begin to set up an infection, 1 mL of the over-night culture of GFP-expressing *S. aureus* in TSB is centrifuged at 18000 ×*g* for 1 min. After removal of the supernatant the bacterial pellet is re-suspended in 1 mL of sterile saline (0.9% w/v NaCl in ddH_2_0) and centrifuged again. Next, the resulting pellet is re-suspended in 1 mL of sterile saline containing 1.25 μm eBioscience^TM^ Cell Proliferation Dye eFluor^TM^ 670 (Thermo Fisher Scientific, cat No. 65-0840-85; eFluor^TM^-670) and incubated at room-temperature for 5 min. Note: eFluor^TM^-670 is diluted into saline from a stock solution immediately prior to use and before re-suspending the washed bacterial pellet. The eFluor^TM^-670 compound is an amine reactive dye that will non-specifically label any protein or structure on the cell surface of *S. aureus* that contains primary amines. After eFluor^TM^-670 labeling the bacterial cells are pelleted (the pellet may now appear blue in color to the naked eye) and the supernatant removed. To quench any unreacted dye and wash the cells the resulting bacterial pellet is re-suspended in 1 mL of bacterial culture media such as TSB, which contains an abundance of protein and peptides, and is incubated for 2 min at room-temperature. After centrifugation, the bacteria are washed once with sterile saline and then re-suspended in 1 mL of serum-free RPMI-1640 (Wisent Inc.) that has been brought to room-temperature. Next the OD600nm of this 1 mL bacterial suspension is measured and is used to generate a 1 mL suspension of bacteria that is diluted to an OD600nm equivalent of 0.5 or ∼4.4 × 10^8^ CFU/mL for wild-type *S. aureus* USA300. For each strain employed, the concentration of cells for an OD600nm of 0.5 should be confirmed as the bacterial cell count is required to set up infections at an appropriate multiplicity of infection (MOI).

For macrophage infections, an MOI of 10 bacteria per macrophage is routinely employed in our laboratory. Due to the voracious capacity of macrophages to phagocytose, an MOI of more than 10 typically results in leukocytes that are engorged with bacteria at very early times after infection. These infections are performed in 12-well tissue culture plates containing ∼6.0 × 10^5^ macrophages per well (setup described below) meaning that 6.0 × 10^6^ bacteria are added per well containing macrophages. To generate a single suspension containing GFP-expressing *S. aureus* USA300 labeled with eFluor^TM^-670, typically, 163 μL of the labeled bacterial suspension at OD600nm 0.5 is added to 12 mL of serum-free RPMI that has been warmed to 37°C. This suspension is used to infect macrophages as described below.

### Growth and Fluorescent Proliferation Dye Labeling of Gram-Negative Bacteria

Gram-negative bacteria including *Citrobacter rodentium*, *Escherichia coli* and *Yersinia pseudotuberculosis* (described in Table 1) were cultured from bacterial glycerol stocks maintained at -80°C. Each bacterium was streaked from a frozen stock onto LB agar plates and grown at 37°C. Next, an isolated colony from each bacterial species was inoculated into 20 mL sterile glass test tubes containing 5 mL of LB broth and grown at 37°C with shaking at 250 rpm overnight. Where appropriate *E. coli* carrying the GFP expression vector pFPV25.1 (Valdivia and Falkow, 1996) was cultured in the presence of ampicillin (100 μg/mL).

To label Gram-negative bacteria, 500 μL of the overnight culture was pelleted by centrifugation and washed 1× with sterile saline [0.9% (w/v) NaCl in ddH_2_O]. The cells were again pelleted and washed with 1 mL of 1× PBS pH 8.0. The resulting cell pellet was then re-suspended in 1 mL PBS pH 8.0 containing 2.5 μm of eBioscience^TM^ Cell Proliferation Dye eFluor^TM^ 670 and incubated for 10 min at room temperature. Technical note: labeling of Gram-negative bacteria was more efficient in PBS at pH 8.0 than in our saline solution. After 10 min, the bacteria were pelleted, washed 1× in sterile LB to quench any unreacted dye, and were then analyzed or used experimentally.

### Macrophage Culture

Routinely, infections in our laboratory are performed in primary murine and human macrophages or immortalized macrophage cell lines.

1. Immortalized macrophage cell lines. For infections using the immortalized murine macrophage-like cell line RAW 264.7 (ATCC^®^ TIB-71^TM^), cells are maintained without antibiotics in RPMI supplemented with 2 mM Glutamine, 25 mM HEPES [4-(2-hydroxyethyl)-1-piperazineethanesulfonic acid] and 5% (v/v) non-heat inactivated fetal bovine serum (Wisent Inc.). HEPES buffer is included in the tissue culture medium so that pH maintenance is relatively insensitive to fluctuations in atmospheric CO_2_. Consequently, the pH of the medium and organellar pH can be maintained in the presence (*i.e.*, within an incubator) or absence (*i.e.*, on the bench top or on the microscope) of constant CO_2_. Antibiotics are purposefully omitted to avoid introducing potentially confounding effects of antibiotic exposure on phagocytosed bacteria as macrophages will regularly consume antibiotic through constitutive pinocytosis (Bohdanowicz et al., 2013; Canton et al., 2016; Flannagan et al., 2016). RAW 264.7 macrophages are routinely grown in T25 vented tissue culture flasks at 37°C in the presence of 5% CO_2_ and humidity of ∼90%. When RAW cells reach ∼70% confluence in a T25 flask, macrophages are split by scraping as per the instructions provided by the ATCC for this cell line. To set up infection assays a 12-well tissue culture plate containing a single sterile 18 mm glass coverslip (Electron Microscopy Sciences, No. 1) in each well is used. Next, a uniform suspension of RAW macrophages is created by adding scraped RAW macrophages, at a density of ∼4.8 × 10^6^ cells per 12 mL of RPMI with FBS. To each well, 1 mL of this suspension (∼4.0 × 10^5^ cells) is added and the plate is incubated overnight (∼ 16 to 18 h) for infection the next day. The RAW cell doubling time is quite fast and so by the following day each well of a 12-well dish will contain RAW macrophages that are ∼55–60% confluent or ∼5.5 to 6.0 × 10^5^ cells per cover glass. Scraping cells to passage them inevitably causes some cell death which accounts for the slightly reduced final number of cells.
2. Primary bone marrow-derived macrophages. In some instances, it may be necessary to perform infections in primary human or murine bone-marrow derived macrophages. Bone-marrow derived macrophages may be particularly useful as this will permit the use of knock-out mice which can allow for investigation into host factors that influence the ability of *S. aureus* to survive and/or replicate intracellularly. For the isolation of bone marrow-derived macrophages we follow standard procedures whereby the murine femur and tibia are flushed into a 50 mL conical tube with sterile 1× PBS and the bone marrow cells collected (Weischenfeldt and Porse, 2008; Davis, 2013). After centrifugation at 500 ×*g* the resulting bone-marrow cell pellet is re-suspended in 3 mL sterile ACK red cell lysis buffer (150 mM NH_4_Cl, 10 mM KHCO_3_, 0.1 mM EDTA, pH 7.4) for 2 min and then diluted 16-fold by addition of sterile 1× PBS. This diluted cell suspension is passed through a 70 μm nylon mesh cell strainer and then subject to centrifugation at 500 × *g* to collect the bone marrow cells. The resulting cell pellet is re-suspended 4 mL sterile serum-free RPMI and the density of viable cells is determined using trypan blue staining. Next, the cells are diluted in RPMI containing 10% (v/v) non-heat inactivated FBS supplemented with 1× pen-strep (Wisent) and recombinant murine M-CSF (10 ng/mL; PeproTech^®^) such that 1.0 × 10^6^ cells are added per well of a 12-well tissue culture dish containing sterile cover glass as described above. The culture medium is replaced on day 2 and day 5 where on day 5 antibiotics are omitted. Macrophages are used for infection between day 7 and day 10 and are infected as described below for RAW macrophages at an MOI of 10 bacteria per macrophage.
3. Primary human macrophages. To derive primary human macrophages peripheral blood monocytes are isolated from healthy volunteers in accordance with protocols approved by the University of Western Ontario Research Ethics Board. Freshly isolated blood is over-laid on Lympholyte-poly cell separation medium (Cedarlane Laboratories) according to the manufacturer’s instructions. In brief, the suspension is centrifuged at 500 ×*g* for 30 min without centrifugal braking. The mononuclear band of cells is removed and transferred to a fresh 50 mL conical tube and topped up to a final volume of 50 mL with sterile 1× PBS. Mononuclear cells are again centrifuged at 500 × *g* for 10 min and the resulting cell pellet is re-suspended in serum-free RPMI. Next, the density of viable cells is determined by trypan blue staining and counting using a hemocytometer. The cells are diluted such that ∼5.0 × 10^5^ cells in a 300 μL volume will be plated into each well of a 12-well tissue culture plate already containing sterile 18 mm glass coverslips. Care is taken to add only 300 μL directly onto each coverglass without causing the cell suspension to run all over the well. This is to ensure maximal recovery of monocytes which preferentially adhere to the glass surface. After 1 h incubation at 37°C in 5% CO_2_ incubator the plate containing adhered monocytes is washed twice with 2 mL sterile 1× PBS per wash. Finally, to each well containing adhered monocytes, 1 mL of RPMI supplemented with 10% (v/v) non-heat inactivated FBS, 1× penicillin-streptomycin solution, and either recombinant human M-CSF for M0 polarized macrophages or recombinant human GM-CSF for M1 polarized macrophages (each at 10 ng/mL, PeproTech^®^). After 5 days at 37°C in a humidified 5% CO_2_ incubator the cells are washed twice with 2 mL sterile 1× PBS and the medium replaced with the same growth medium, however, antibiotics are omitted. In addition, the medium to derive M1 polarized macrophages is supplemented recombinant human interferon-γ (10 ng/mL, PeproTech^®^) and 250 ng/mL lipopolysaccharide (Santa Cruz Biotechnology^®^). Alternatively, one can derive M2 polarized macrophages at this time by adding to the M-CSF containing medium interleukin-4 (10 ng/mL, PeproTech^®^). At day 7 to 10 the macrophages are ready to be used for phagocytosis of *S. aureus* which is performed as described below using an MOI of 10 bacteria per macrophage.

### Infection Assays

To infect these cells 1 mL of the diluted suspension of GFP-expressing eFluor^TM^-670 labeled *S. aureus* (described above) is added to each well of a 12-well plate containing macrophages. To synchronize phagocytosis the plate is centrifuged immediately at 277 ×*g* for 2 min at room temperature and then incubated at 37°C in the presence of 5% CO_2_ for 30 min. We prefer to synchronize phagocytosis by centrifugation without exposing *S. aureus* or macrophages to the shock of 4°C to avoid untoward effects of cold exposure such as de-polymerization of macrophage microtubules (Breton and Brown, 1998; Lodish et al., 2000) as microtubules can contribute to phagocytosis (Allen and Aderem, 1996; Gilberti and Knecht, 2015). After 30 min the media containing bacteria is aspirated and replaced with warmed serum-free RPMI containing gentamicin (100 μg/mL) for 1 h. After incubation with gentamicin each well containing macrophages is washed twice with a 2-mL volume of sterile 1× PBS each time and then incubated further with warmed RPMI supplemented with 5% (v/v) FBS without gentamicin at 37°C in 5% CO_2_.

For infection of RAW 264.7 macrophages with GFP-expressing *E. coli* ML35 bacteria, the RAW cells were prepared as described above. Infections were also performed exactly as described for *S. aureus*, however, *E. coli* was added to RAW macrophages at an MOI of 100 because this strain is non-pathogenic.

### Fluorescent Staining of Macrophages

In many instances, it is desirable to characterize the subcellular niche in which phagocytosed bacteria reside. Routinely, when investigating the interaction of *Staphylococci* with macrophages we employ a variety of fluorescence-based staining that is compatible with eFluor^TM^-670 labeling of *S. aureus*. Different stains and fluorescent markers are used for delineation of the cell membrane and subcellular compartments, as described below.

1. Plasmalemma staining. To simply delineate the plasmalemma of the macrophages and label extracellular cocci just prior to macrophage fixation, wheat germ agglutinin (WGA) conjugated with tetramethylrhodamine (TMR) is diluted in 1× PBS at a concentration of 1 μg/mL and incubated for 2 min at room temperature prior to rinsing twice with 1 mL of PBS. After rinsing, TMR-WGA stained cells are fixed with 4% (v/v) PFA for 20 min prior to rinsing with 1× PBS and fluorescence imaging.
2. Phagolysosome staining. To determine whether phagocytosed *S. aureus* reside within mature, acidic phagolysosomes a variety of procedures can be performed, however, we routinely perform Lysotracker^TM^ staining and immunostaining of host cell lysosome-associated membrane protein-1 (LAMP-1).
2a. Lysotracker^TM^ Staining. For Lysotracker^TM^ staining macrophages already infected with *S. aureus* are incubated with 250 nM Lysotracker^TM^ probe diluted in serum-free RPMI for a total of 5 to 7 min prior to washing away the probe. Macrophages are then imaged immediately while alive to avoid the introduction of artifacts through fixation and/or immunostaining. Live imaging of cells can be done with the cells bathed in either RPMI containing 25 mM HEPES or alternatively live cell imaging buffer (150 mM NaCl, 5 mM KCl, 1 mM MgCl_2_, 100 μm EGTA, 2 mM CaCl_2_, and 20 mM HEPES, pH 7.4) can be used. Lysotracker^TM^ staining can be performed at any time during an infection, however, staining should always be performed immediately before imaging that specific time point.
2b. LAMP-1 Immunostaining. To analyze the cellular distribution of LAMP-1 with respect to the *S. aureus*- containing phagosome we routinely perform immunostaining of either murine or human macrophages to detect endogenous LAMP-1. Here, macrophages are first fixed with 4% (v/v) paraformaldehyde for 20 min at room temperature prior to permeabilization of PFA-fixed cells with 100% methanol pre-chilled to -20°C. While methanol can also be used in some instances for fixation, methanol is not compatible with GFP, and if used without PFA fixation first will distort the GFP chromophore and compromise fluorescence. After 3 min incubation at -20°C the fixed and permeabilized cells are washed once with 1× PBS and then incubated for at least 3 h in 1 mL of human plasma from primary macrophage isolations that is kept frozen. Due to the presence of protein A on the surface of *S. aureus*, human plasma is used to block non-specific protein A binding of anti-LAMP-1 or secondary antibodies as it contains milligram quantities of IgG per mL. If the bacterium under investigation does not express protein A or another IgG-binding protein, blocking can be done in 5% (w/v) skim milk reconstituted in 1× PBS instead of human plasma. After blocking, detection of murine LAMP-1 is through rat-anti-mouse LAMP-1 antibody (clone 1D4B) whereas detection of human LAMP-1 requires use of mouse-anti-human LAMP-1 antibody (clone H4A3). Anti-LAMP-1 antibodies are purchased as supernatant from the Developmental Studies Hybridoma Bank (DSHB) and H4A3 was deposited to the DSHB by J. T. August and J. E. K. Hildreth and 1D4B by J. T. August. To stain LAMP-1 the appropriate antibody clone is diluted 1:100 in PBS containing 50% (v/v) human plasma and incubated for 1 h at room temperature. After serially washing the cells, primary antibody binding is detected by 45 min incubation with secondary goat anti-rat or goat anti-mouse Cy^TM^3-conjugated antibody (Jackson Immunoresearch) diluted in PBS containing 50% (v/v) human plasma as appropriate. Cy^TM^3-conjugated secondary antibodies are employed as the spectra of this dye is compatible with GFP (expressed by *S. aureus*) and far-red Fluor^TM^-670 (labeling un-replicated bacteria).

### Fluorescence Microscopy

Wide-field fluorescence microscopy is performed on a Leica DMI6000 B inverted microscope equipped with 40× (NA 1.3), 63× (NA 1.4), and 100× (NA 1.4) oil immersion PL-Apo objectives, a Leica 100-W Hg high-pressure light source, and a Photometrics Evolve 512 Delta EM-CCD camera. The EM-CCD camera provides superior sensitivity for photon detection. This microscope is also outfitted with an objective warmer and an enclosed heated stage insert with CO_2_ perfusion (Live Cell Instruments). This microscope is equipped with the ET-Sedat-quad 89000 series excitation and emission filter set (Chroma Technologies) for DAPI (4′,6-diamidino-2-phenylindole), GFP/FITC, Cy3/Alexa 555, and Cy5/Alexa 647 imaging. For live-cell imaging, coverslips carrying macrophages were placed in a magnetic imaging chamber and bathed in SF-RPMI buffered with Na bicarbonate and 25 mM HEPES. Live-cell imaging employed a heated stage and objective warmer set to 37°C and in the presence of 5% CO_2_ as necessary.

Images were acquired as z-series using the Leica LAS X microscope software package. In FIJI (Schindelin et al., 2012) the raw imaging data for a given series were Z projected under the stacks feature where the projected image is the sum of three consecutive slices. After the Z projection tool is used, images are split into individual channels, contrast enhanced and cropped prior to image overlaying/merging. Importantly, the gamma is never altered, and fluorescence intensity measurements, if made, are only done on raw data with background subtraction.

### Ethics Statement

Blood was obtained, with informed and written permission, only from healthy adult volunteers, in compliance with protocol 109059 approved by the Office of Research Ethics at the University of Western Ontario. All animal protocols (protocol 2017-028) were reviewed and approved by the University of Western Ontario Animal Use Subcommittee, a subcommittee of the University Council on Animal Care. Protocols adhered to guidelines set out by the Canadian Council on Animal Care.

## Results

### Detection of Replicating Intracellular Bacteria

**FIGURE 2:**
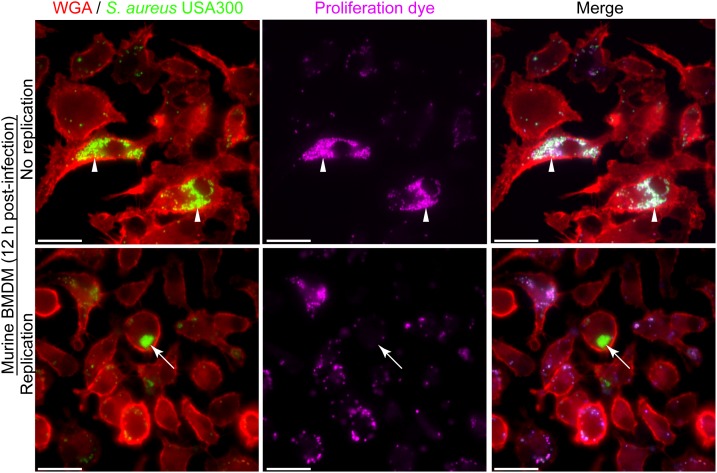
The presence of large populations of intracellular bacteria does not accurately demonstrate the presence of replicating bacteria. The representative images are of TMR-WGA labeled murine bone-marrow-derived macrophages (BMDM, in red) that were incubated with GFP-expressing *S. aureus* USA300 (in green) labeled with eFluor^TM^-670 (pseudo-colored pink) at an MOI of 10. Images were acquired at 12 h post-infection. Arrow heads in the top panels indicate macrophages containing many *S. aureus* cocci, however, these bacteria are eFluor^TM^-670 positive and therefore have not replicated. White arrows point to GFP-positive yet eFluor^TM^-670 negative *S. aureus* indicating these bacteria have indeed replicated intracellularly. These data represent observations made from more than six independent experiments. Scale bar is ∼10 μm.

**FIGURE 3:**
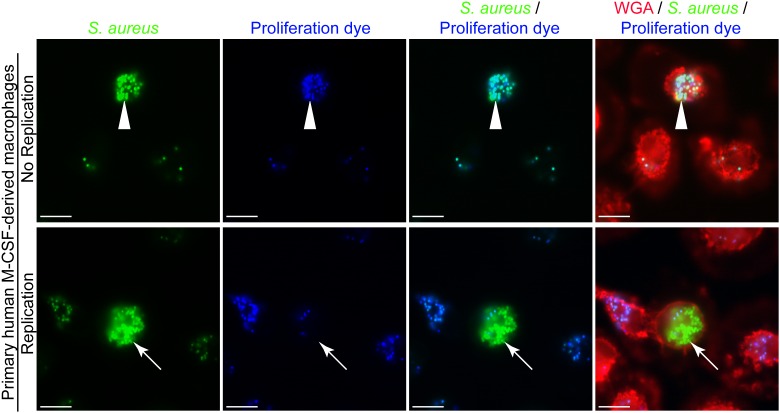
Heterogeneity in the number of intracellular bacteria occurs in primary human M-CSF-derived macrophages. Primary human M-CSF-derived macrophages derived from an otherwise healthy donor were infected with *S. aureus* after 7 days of differentiation. Macrophages are marked with TMR-WGA (in red) while *S. aureus* USA300 is expressing GFP (in green). At the outset of the infection bacteria were co-labeled with eFluor^TM^-670 proliferation dye (pseudo-colored blue). These representative images were taken at 12 h post-infection and represent common events from multiple (>6) independent experiments. The white arrow head points to a primary phagocyte containing many cocci while the surrounding cells contain few. These bacteria are also marked with eFluor^TM^-670 proliferation dye at this time-point. White arrows point to a phagocyte that contains many GFP-positive cocci that are eFluor^TM^-negative indicating these bacteria have replicated. In contrast the surrounding cells contain few GFP-positive cocci that are also eFluor^TM^-670-positive. Scale bar is ∼10 μm.

The phagocytic and microbicidal capacity of macrophages can be heterogeneous complicating the detection of intracellular replicating *S. aureus*. The need to accurately identify intracellularly proliferating bacteria underpins our ability to understand host-pathogen interactions at the cellular level. Through our investigation of the interaction between macrophages and methicillin-resistant *S. aureus* strain USA300 we have realized that accurately identifying replicating bacteria within the host cell can be complicated. This is, in part, due to heterogeneity between macrophages in terms of the number of bacteria that might reside in any given phagocyte despite the use of a single multiplicity of infection. To illustrate this, phagocytosis assays of GFP-expressing *S. aureus* strain USA300 that is co-labeled with the proliferation dye eFluor^TM^-670 were performed using murine bone marrow derived macrophages (BMDMs) (Figure 2). Here, at 12 h post-infection it is evident that every BMDM can ingest *S. aureus* to some extent, however, there is significant variation in the number of GFP-positive cocci that reside in each macrophage. In the absence of the proliferation dye, it might be concluded that some of the macrophages in the top panel contain replicating *S. aureus* because they contain many more cocci at 12 h post-infection as compared to the surrounding phagocytes (Figure 2, top panels). However, through co-labeling GFP-expressing *S. aureus* with eFluor^TM^-670 it is evident that these intracellular cocci have not replicated as they remain proliferation dye positive. In contrast, at the same time point there exist instances whereby macrophages contain intracellular GFP-positive *S. aureus* cocci that are devoid of the proliferation dye eFluor^TM^-670 indicating these bacteria grew within the macrophage (Figure 2, bottom panels). Importantly, the heterogeneity with which macrophages ingest *S. aureus* or continue to ingest cocci off the cover glass occurs not only in primary murine macrophages but is also observed when phagocytosis assays are performed using primary human M-CSF-derived macrophages (Figure 3).

### Loss of Proliferation Dye Fluorescence Requires Bacterial Replication

**FIGURE 4:**
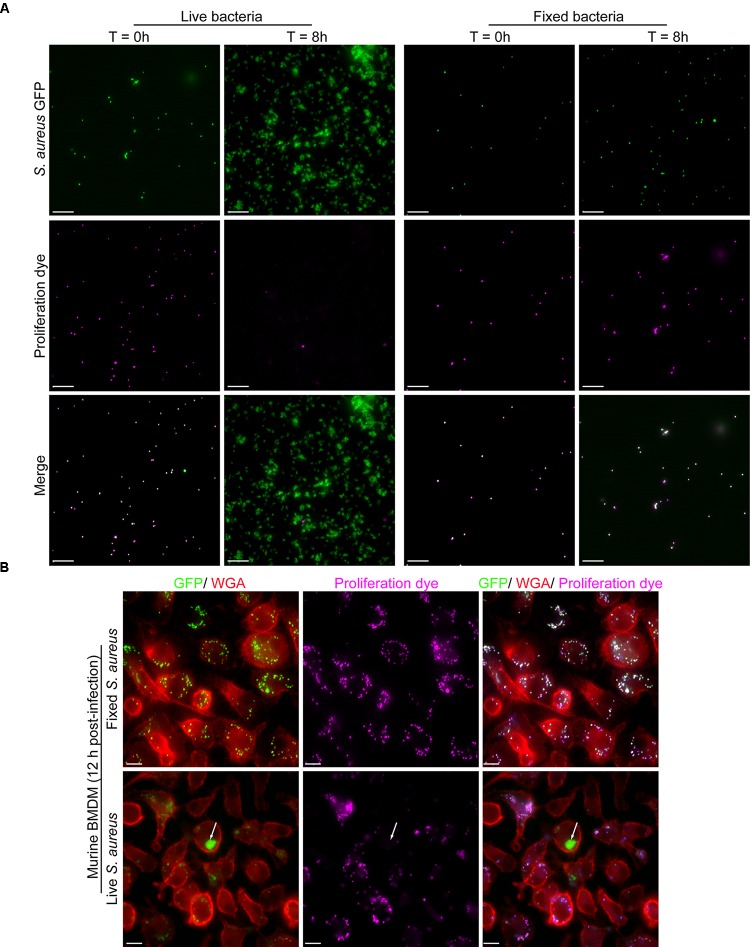
Proliferation dye can accurately identify bacteria that have grown *in vitro* and within murine bone marrow-derived macrophages. In **(A)** GFP-expressing *S. aureus* were co-labeled with eFluor^TM^-670 proliferation as described in Materials and Methods. In addition, some of these cells were inactivated by PFA fixation. After inoculation of TSB images of the live and fixed bacteria were acquired at time 0 and 8 h post-inoculation. Scale bar is ∼10 μm. In **(B)** murine bone marrow-derived macrophages (BMDM) were infected with live and fixed GFP-expressing *S. aureus* that were marked with eFluor^TM^-670 proliferation dye. Macrophages in red were labeled with TMR-WGA right before fixation. These representative images of macrophages containing fixed (top row) or live (bottom row) *S. aureus* were acquired at 12 h post-infection. In the top row, all GFP-positive cocci are eFluor^TM^-670 positive whereas in the bottom row at 12 post-infection GFP-positive yet proliferation dye negative bacteria can be detected (white arrow). These data represent observations from more than ten independent experiments. Scale bar is ∼10 μm.

Again, the proliferation assay we describe functions akin to lymphocyte proliferation assays using carboxyfluorescein diacetate succinimidyl ester (CFSE) where labeled lymphocytes initially appear intensely fluorescent, however, through successive cell division become increasingly weaker for the CFSE signal (Quah et al., 2007). Instead of CFSE we have employed eFluor^TM^-670 and conjugate this fluorescent probe to *S. aureus* bacteria also expressing GFP. To demonstrate the utility of the eFluor^TM^-670 dye for the detection of proliferating bacteria we performed a series of experiments to demonstrate that only living bacteria that can undergo cell division become proliferation dye negative over time. To this end we labeled live GFP-expressing *S. aureus* USA300 with eFluor^TM^-670 exactly as described in materials and methods. These bacteria were then either left untreated or subject to paraformaldehyde fixation to inactivate the bacteria and render them unable to proliferate. Next, these live and inactivated bacteria that were positive for both GFP and eFluor^TM^-670 were inoculated into TSB and cultured for 8 h at 37°C. At the start of this experiment samples of each culture were taken and imaged by fluorescence microscopy which revealed that at the outset all GFP-positive bacteria were indeed positive for far-red eFluor^TM^-670 fluorescence (Figure 4A). Importantly, by 8 h post-inoculation GFP-positive yet eFluor^TM^-670 negative bacteria were only evident in the “active” culture where *S. aureus* could undergo cell division (Figure 4A). These observations were entirely consistent with the notion that *S. aureus* labeled with eFluor^TM^-670 could only become negative for the proliferation dye through bacterial replication. From this analysis, it is evident that loss of eFluor^TM^-670 fluorescence occurs through division of *S. aureus* cocci.

To demonstrate that this also transpires intracellularly we performed macrophage infections using primary murine bone marrow-derived macrophages. Here macrophages were incubated with live GFP-expressing *S. aureus* USA300 or fixed GFP-positive *S. aureus* that were also eFluor^TM^-670-positive. Here at 12 h post-infection only eFluor-negative bacteria can be detected for live *S. aureus* whereas all fixed bacteria, at this time point, maintained the proliferation dye (Figure 4B). Taken together these data reveal that in the phagolysosome of macrophages the eFluor^TM^-670 proliferation dye is stable and that only replicating cells can become negative for the dye long after infection.

### Fluorescence-Based Proliferation Analysis Is Compatible With Additional Phagosomal Fluorescent Probes

**FIGURE 5:**
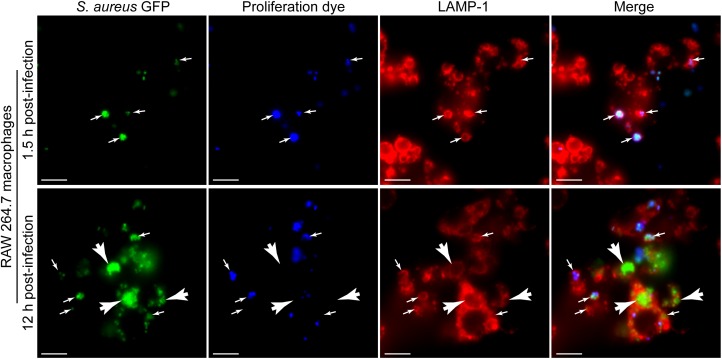
Fluorescence proliferation dye can be used to identify bacteria replicating in lysosome associated membrane protein-1 (LAMP-1) positive compartments. The representative images depict RAW 264.7 macrophages infected with GFP-expressing *S. aureus* USA300 (in green) co-labeled with eFluor^TM^-670 (in blue) that are also immune-stained for endogenous LAMP-1 protein (in red). Images are of cells at 1.5 and 12 h post-infection and are representative of data normally obtained from greater than ten independent experiments. Small white arrows point to GFP-positive cocci that are eFluor^TM^-670 positive and that are demarcated by LAMP-1 fluorescence. White arrow heads point to GFP-positive yet proliferation dye negative *S. aureus* that are LAMP-1 positive. Scale bar equals 10 μm. Note: The image series shown for the 12 h time point is presented elsewhere in an alternate form (Copyright © American Society for Microbiology; Flannagan et al., 2018a).

To demonstrate the utility of the eFluor^TM^-670 dye for the identification of *S. aureus* growing within macrophages in the context of additional cellular probes we first performed macrophage infections in parallel with LAMP-1 immunofluorescence. Previously we have demonstrated that *S. aureus* fails to replicate immediately after phagocytosis and instead commences proliferating hours after infection (Flannagan et al., 2016). Consistent with these earlier observations, we find again that at 1.5 h post-infection, all *S. aureus* cocci are GFP and eFluor^TM^-670 positive indicating they have not proliferated (Figure 5, top panels). In contrast, by 12 h post-infection GFP-positive cocci that are eFluor^TM^-670 negative are readily detectable and easily distinguished from those bacteria that remain eFluor^TM^-670 positive (Figure 5, bottom panels). In addition, through LAMP-1 immunostaining of these samples it is evident that *S. aureus*, even when replicating (i.e., GFP-positive yet eFluor^TM^-670 negative), can be confined to LAMP-1 positive compartments (Figure 5).

**FIGURE 6:**
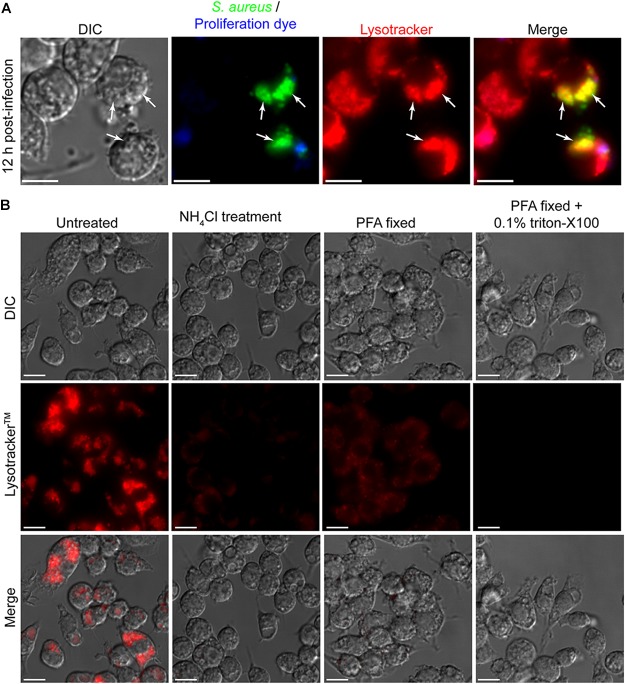
Cellular probes such as Lysotracker^TM^ Red DND-99 are compatible with the fluorescent reporter of proliferation dye. In **(A)** RAW 264.7 macrophages containing live *S. aureus* were stained with Lysotracker^TM^ and imaged by live cell fluorescence microscopy. At the outset of infection GFP-expressing bacteria were labeled with eFluor^TM^-670 and here at 12 h post-infection GFP-positive yet eFluor^TM^-670 negative bacteria can be detected (white arrows). These bacteria also co-localize with Lysotracker^TM^. Scale bar is ∼10 μm. In **(B)** RAW macrophages that were uninfected were stained with Lysotracker^TM^ and imaged either live, after fixation, or after fixation and permeabilization. These representative images were acquired under the same acquisition parameters to allow for direct comparison of fluorescence intensities and represent observations made from more than six independent experiments. In one instance, live RAW cells were first treated with 40 mM of the weak base NH_4_Cl prior to lysotracker staining. Scale bars equal ∼10 μm.

To further investigate the niche in which *S. aureus* is replicating it is desirable to employ additional fluorescent probes such as Lysotracker^TM^ Red DND-99. With these additional probes, important information about the interaction of the replicating pathogen with the host cell can be gained. To demonstrate the compatibility of Lysotracker^TM^ staining with the eFluor^TM^-670 fluorescence-based proliferation assay we performed macrophage infections where at 12 h post-infection phagocytes were also stained with Lysotracker^TM^ staining (Figure 6A). From this procedure it is evident that GFP-expressing *S. aureus* that are eFluor-negative can be detected within Lysotracker^TM^-positive vacuoles (Figure 6A). Here we perform Lysotracker^TM^ staining only on live cells and image immediately after staining. It is important to consider how the Lysotracker^TM^ probe is being used and how stained cells are processed for imaging. This is done to limit artifacts that can be introduced by fixation and or permeabilization. Indeed, Lysotracker^TM^ is not a fixable probe and its retention and cellular distribution is sensitive to fixation and/or permeabilization of cells after staining. To demonstrate this, we performed image analysis of Lysotracker^TM^ stained RAW macrophages that were not manipulated, were pre-treated with the weak base ammonium chloride, or were fixed and or permeabilized after lysotracker staining (Figure 6B). As expected when stained immediately prior to imaging while alive it is evident that RAW macrophages possess a profusion of acidic compartments that robustly accumulate the acidotropic dye (Figure 6B). In contrast, when macrophages are pre-treated with 40 mM NH_4_Cl for 10 min prior to Lysotracker^TM^ staining and live cell imaging, it is evident that accumulation of the probe is severely compromised (Figure 6B). When 20 min fixation with paraformaldehyde is performed immediately after Lysotracker^TM^ staining and right before imaging it is evident that the cellular distribution of Lysotracker^TM^ differs from living cells despite there being retention of fluorescence. For instance, in live cells Lysotracker^TM^ prominently accumulates within large spacious vacuoles present with the macrophage cytoplasm, however, in fixed cells these vacuolar structures are devoid of the Lysotracker^TM^ probe (Figure 6B). Moreover, when permeabilization is performed on Lysotracker^TM^ stained cells as would be done to detect phagocytosed bacteria or host proteins through antibody staining, the Lysotracker^TM^ probe is lost entirely from the cell. Taken together these data reveal that Lysotracker^TM^ staining is compatible with the eFluor^TM^-670 fluorescent proliferation dye, however, care should be taken to avoid fixation and/or permeabilization.

### Fluorescence-Based Proliferation Assays Can Be Employed to Analyze the Growth of Gram-Negative Bacteria

**FIGURE 7:**
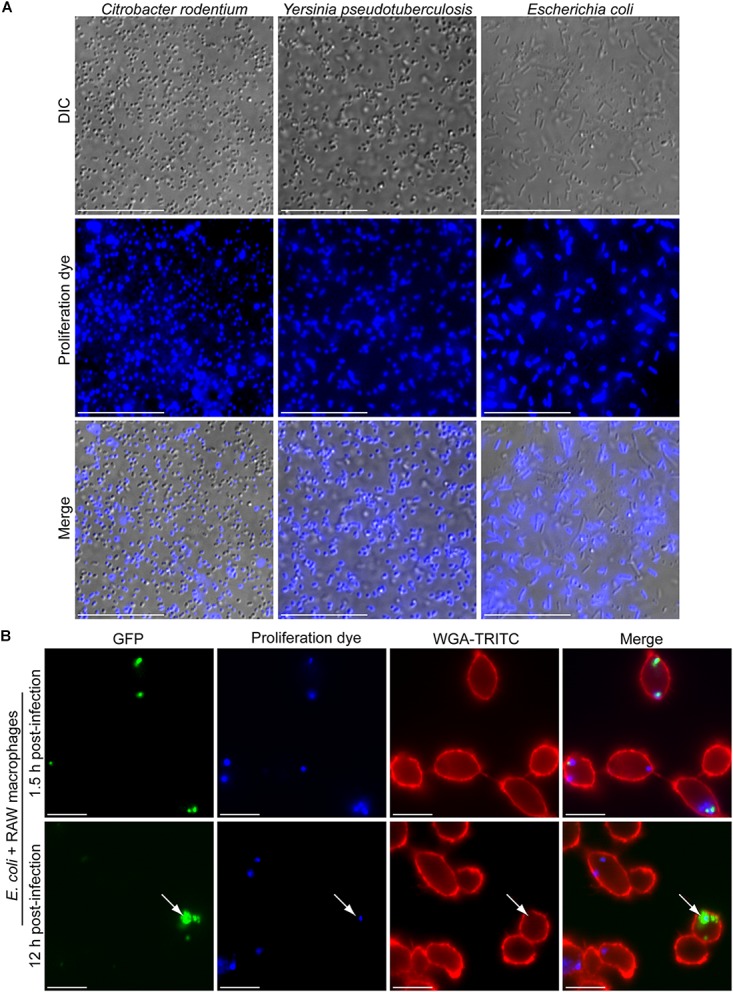
Gram-negative bacteria are amenable to fluorescence-based proliferation assays. In **(A)** the micrographs depict *C. rodentium*, *Y. pseudotuberculosis*, and *E. coli* DH5α bacteria that were incubated with eFluor^TM^-670 proliferation dye for a period of 10 min. In **(B)** the fluorescent micrographs depict intracellular *E. coli* ML35 expressing GFP (in green) that are co-labeled with the eFluor^TM^-670 (in blue). At 1.5 h post-infection (top row) and 12 h post-infection (bottom row) eFluor^TM^-670 positive bacteria can be detected. The white arrow points to a rare instance where GFP-positive yet eFluor^TM^-670-negative *E. coli* could be detected within RAW macrophages marked with TRITC-WGA (in red). These micrographs are representative of two independent experiments. Scale bars are ∼10 μm.

Based on the amine reactivity of the eFluor^TM^-670 dye we predicted that this probe could also be used to mark and identify replicating Gram-negative bacteria. To test this notion, we utilized select Gram-negative bacteria including *Y. pseudotuberculosis*, *C. rodentium*, and *E. coli* DH5a and tested whether we could detect the eFluor^TM^-670 dye in association with the bacteria by fluorescence microscopy. Initial attempts to label these bacteria following the protocol we described for *S. aureus* revealed that while the dye could indeed label some of the bacteria many bacterial cells remained unmarked (data not shown). Clearly the protocol that we employed for *S. aureus* was less efficient for these Gram-negative bacteria. To overcome this, we performed eFluor^TM^-670 labeling in sterile 1× PBS set at pH 8.0 as this buffer has been previously employed to react *N*-hydroxysuccinimidobiotin with *Y. pseudotuberculosis* bacteria (Sarantis et al., 2012). Using this buffer, it was evident that *C. rodentium*, *Y. pseudotuberculosis*, and *E. coli* DH5α could be labeled with eFluor^TM^-670 proliferation dye under the conditions employed here (Figure 7A). Interestingly, the extent to which each bacterial species labeled appeared to differ as some *C. rodentium* and primarily elongated *E. coli* DH5α cells appeared to remain refractory to the dye. In contrast, virtually every *Y. pseudotuberculosis* bacillus was marked with eFluor^TM^-670 (Figure 7A). Nevertheless, it was evident from these experiments that Gram-negative bacteria could in principle be labeled with the proliferation dye, however, optimization of this procedure would have to be performed for each genus, species and strain being analyzed.

Next, we tested whether we could detect eFluor^TM^-670 dye on Gram-negative bacteria having been phagocytosed by macrophages and whether the loss of the dye from the bacteria could inform us on whether the bacteria had replicated. To this end we employed *E. coli* ML35 carrying the GFP expression vector pFPV25.1 and performed phagocytosis assays using RAW macrophages after labeling the bacteria with eFluor^TM^-670 as described above. *E. coli* ML35 has previously been shown to label with eFluor^TM^-670 proliferation dye and has been used in a heat-inactivated form for phagocytosis assays (Yin et al., 2016). Using live *E. coli* ML35 it was evident that at 1.5 h post-infection the *E. coli* bacteria could be detected within RAW macrophages and were GFP and eFluor^TM^-670 positive (Figure 7B). Interestingly, at 12 h post-infection many eFluor^TM^-670-positive *E. coli* appeared GFP-negative or were poorly GFP-fluorescent, consistent with intracellular killing of these bacteria. Despite this, some instances where GFP-bright *E. coli* that were now devoid of eFluor^TM^-670 fluorescence could be observed indicating these bacteria have replicated (Figure 7B). In support of this, these eFluor^TM^-negative *E. coli*, were also found as GFP-positive foci within macrophages, indicating they represent a nidus of replicating bacteria.

## Discussion

Here we present a fluorescence-based method for the accurate identification of *S. aureus* bacteria that can replicate within host cells. Moreover, because this assay is compatible with additional cellular probes (i.e., Lysotracker^TM^ and LAMP-1 immunofluorescence) it is possible to identify and characterize the subcellular niches in which *S. aureus* proliferates. To date, we have employed this assay to characterize the replication of *S. aureus* and *S. lugdunensis* within macrophages (Flannagan et al., 2016, 2018a,b), however, this assay should be easily applied to the study of many bacterial pathogens including Gram-positive and Gram-negative bacteria. In support of this we have demonstrated that at least some Gram-negative bacteria can be labeled and that proliferation assays can be performed whilst *E. coli*, for example, is inside RAW macrophages. The utility of this dye for Gram-negative bacteria is perhaps not surprising considering the fact that eFluor^TM^-670 reacts with primary amines on the bacterial cell surface and *N*-hydroxysuccinimide esters, which also react with primary amines, have been employed to chemically modify the cell surface of Gram-negative bacteria such as *Y. pseudotuberculosis* and *Burkholderia cenocepacia* (Flannagan et al., 2012; Sarantis et al., 2012). Nevertheless, optimization for labeling distinct bacterial species and/or strains will be necessary.

The fluorescent proliferation assay presented here employed GFP-expressing *S. aureus*, however, from our use of additional probes (i.e., Lysotracker^TM^ Red DND-99 and Cy^TM^3 fluorescence) it should be evident that the spectra of the eFluor^TM^-670 probe is compatible with both red and green fluorophores. With this versatility, experiments can be performed with bacteria that express red fluorescent proteins such as mCherry or mRFP and will still be compatible with cellular probes with spectra comparable to GFP/FITC (e.g., FITC-dextran). In addition, the images presented here were acquired by wide-field fluorescence microscopy with an EM-CCD camera meaning this analysis does not require the use of confocal microscopy. Nevertheless, consideration must be given to the use of appropriate excitation and emission filter sets and the excitation and emission spectra of the probes being used.

The proliferation assay has been a powerful tool that has enabled the characterization of intracellular *Staphylococci* (Flannagan et al., 2016, 2018a,b), however, it is important to note that there are limitations. For instance, we have found that this proliferation analysis is best suited for fluorescence microscopy where individual cells can be visualized and analyzed. Attempts to accurately assess bacterial proliferation by flow cytometry have failed. This is because macrophages can contain multiple phagosomes where the bacteria may or may not be replicating. For instance, a macrophage having ingested several cocci which reside in three different phagosomes may at 12 h post-infection have bacteria replicating in only one of those phagosomes. If analyzed by flow cytometry this cell would be detected as GFP and eFluor^TM^-positive and the observation that one phagosome was permissive to bacterial growth would be lost. Furthermore, sorting cells based on GFP intensity (i.e., GFP bright cells at 12 h would contain replicating bacteria) may erroneously exaggerate the extent to which the bacteria have replicated. This is illustrated in Figure 2 which was why we began to employ this assay using the eFluor^TM^-670 dye. Regardless of these limitations we believe this assay is a powerful tool that allows for the identification replicating bacteria and, when used in parallel with other cellular probes allows for the identification and characterization of the subcellular niche in which bacterial replication occurs.

In summary, the proliferation assay presented here is a useful tool that enables accurate identification of intracellularly growing bacteria. Moreover, through additional cellular probes, important information pertaining to the intracellular niche in which bacteria replicate can be obtained. Despite this, caution should be exercised to ensure that probes such as Lysotracker^TM^ are used appropriately.

## Author Contributions

All authors contributed significantly to this work. RF conducted each experiment, analyzed the data, and prepared the manuscript. DH analyzed the data and edited the manuscript.

## Conflict of Interest Statement

The authors declare that the research was conducted in the absence of any commercial or financial relationships that could be construed as a potential conflict of interest.

## References

[B1] Allen L. A. Aderem A. (1996). Molecular definition of distinct cytoskeletal structures involved in complement- and Fc receptor-mediated phagocytosis in macrophages. *J. Exp. Med.* 184 627–637. 10.1084/jem.184.2.627 8760816 PMC2192718

[B2] Bohdanowicz M. Schlam D. Hermansson M. Rizzuti D. Fairn G. D. Ueyama T. (2013). Phosphatidic acid is required for the constitutive ruffling and macropinocytosis of phagocytes. *Mol. Biol. Cell* 24 1700–1712. 10.1091/mbc.E12-11-0789 23576545 PMC3667723

[B3] Breton S. Brown D. (1998). Cold-induced microtubule disruption and relocalization of membrane proteins in kidney epithelial cells. *J. Am. Soc. Nephrol.* 9 155–166. 9527391 10.1681/ASN.V92155

[B4] Canton J. Schlam D. Breuer C. Gütschow M. Glogauer M. Grinstein S. (2016). Calcium-sensing receptors signal constitutive macropinocytosis and facilitate the uptake of NOD2 ligands in macrophages. *Nat. Commun.* 7:11284. 10.1038/ncomms11284 27050483 PMC4823870

[B5] Davis B. K. (2013). Isolation, culture, and functional evaluation of bone marrow-derived macrophages. *Methods Mol. Biol.* 1031 27–35. 10.1007/978-1-62703-481-4-3 23824883

[B6] Edwards A. M. Massey R. C. (2011). Invasion of human cells by a bacterial pathogen. *J. Vis. Exp.* 49:2693. 10.3791/2693 21445052 PMC3339868

[B7] Fairn G. D. Grinstein S. (2012). How nascent phagosomes mature to become phagolysosomes. *Trends Immunol.* 33 397–405. 10.1016/j.it.2012.03.003 22560866

[B8] Flannagan R. S. Cosío G. Grinstein S. (2009). Antimicrobial mechanisms of phagocytes and bacterial evasion strategies. *Nat. Rev. Microbiol.* 7 355–366. 10.1038/nrmicro2128 19369951

[B9] Flannagan R. S. Heit B. Heinrichs D. E. (2016). Intracellular replication of *Staphylococcus aureus* in mature phagolysosomes in macrophages precedes host cell death, and bacterial escape and dissemination. *Cell. Microbiol.* 18 514–535. 10.1111/cmi.12527 26408990

[B10] Flannagan R. S. Jaumouillé V. Huynh K. K. Plumb J. D. Downey G. P. Valvano M. A. (2012). *Burkholderia cenocepacia* disrupts host cell actin cytoskeleton by inactivating Rac and Cdc42. *Cell. Microbiol.* 14 239–254. 10.1111/j.1462-5822.2011.01715.x 22023324

[B11] Flannagan R. S. Kuiack R. C. McGavin M. J. Heinrichs D. E. (2018a). *Staphylococcus aureus* uses the GraXRS regulatory system to sense and adapt to the acidified phagolysosome in macrophages. *mBio* 9:e01143-18. 10.1128/mBio.01143-18 30018109 PMC6050959

[B12] Flannagan R. S. Watson D. W. Surewaard B. G. J. Kubes P. Heinrichs D. E. (2018b). The surreptitious survival of the emerging pathogen *Staphylococcus lugdunensis* within macrophages as an immune evasion strategy. *Cell. Microbiol.* 20:e12869. 10.1111/cmi.12869 29904997

[B13] Flannagan R. S. Heit B. Heinrichs D. E. (2015). Antimicrobial mechanisms of macrophages and the immune evasion strategies of *Staphylococcus aureus*. *Pathogens* 4 826–868. 10.3390/pathogens4040826 26633519 PMC4693167

[B14] Gilberti R. M. Knecht D. A. (2015). Macrophages phagocytose nonopsonized silica particles using a unique microtubule-dependent pathway. *Mol. Biol. Cell.* 26 518–529. 10.1091/mbc.E14-08-1301 25428990 PMC4310742

[B15] Gries C. M. Sadykov M. R. Bulock L. L. Chaudhari S. S. Thomas V. C. Bose J. L. (2016). Potassium uptake modulates *Staphylococcus aureus* metabolism. *mSphere* 1:e0125-16. 10.1128/mSphere.00125-16 27340697 PMC4911797

[B16] Jubrail J. Morris P. Bewley M. A. Stoneham S. Johnston S. A. Foster S. J. (2015). Inability to sustain intraphagolysosomal killing of *Staphylococcus aureus* predisposes to bacterial persistence in macrophages. *Cell. Microbiol.* 18 80–96. 10.1111/cmi.12485 26248337 PMC4778410

[B17] Lodish H. Berk A. Zipursky S. L. Matsudaira P. Baltimore D. Darnell J. (2000). *Molecular Cell Biology*, 4th Edn Available at: https://www.ncbi.nlm.nih.gov/books/NBK21522/

[B18] Portnoy D. A. Auerbuch V. Glomski I. J. (2002). The cell biology of *Listeria monocytogenes* infection: the intersection of bacterial pathogenesis and cell-mediated immunity. *J. Cell Biol.* 158 409–414. 10.1083/jcb.200205009 12163465 PMC2173830

[B19] Portnoy D. A. Jacks P. S. Hinrichs D. J. (1988). Role of hemolysin for the intracellular growth of *Listeria monocytogenes*. *J. Exp. Med.* 167 1459–1471. 10.1084/jem.167.4.1459 2833557 PMC2188911

[B20] Quah B. J. C. Warren H. S. Parish C. R. (2007). Monitoring lymphocyte proliferation in vitro and in vivo with the intracellular fluorescent dye carboxyfluorescein diacetate succinimidyl ester. *Nat. Protoc.* 2 2049–2056. 10.1038/nprot.2007.296 17853860

[B21] Sarantis H. Balkin D. M. De Camilli P. Isberg R. R. Brumell J. H. Grinstein S. (2012). Yersinia entry into host cells requires Rab5-dependent dephosphorylation of PI(4,5)P 2 and membrane scission. *Cell Host Microbe* 11 117–128. 10.1016/j.chom.2012.01.010 22341461 PMC4489550

[B22] Sarantis H. Grinstein S. (2012). Subversion of phagocytosis for pathogen survival. *Cell Host Microbe* 12 419–431. 10.1016/j.chom.2012.09.001 23084912

[B23] Sayedyahossein S. Xu S. X. Rudkouskaya A. McGavin M. J. McCormick J. K. Dagnino L. (2015). *Staphylococcus aureus* keratinocyte invasion is mediated by integrin-linked kinase and Rac1. *FASEB J.* 29 711–723. 10.1096/fj.14-262774 25416549

[B24] Schindelin J. Arganda-Carreras I. Frise E. Kaynig V. Longair M. Pietzsch T. (2012). Fiji: an open-source platform for biological-image analysis. *Nat. Methods* 9 676–682. 10.1038/nmeth.2019 22743772 PMC3855844

[B25] Tranchemontagne Z. R. Camire R. B. O’Donnell V. J. Baugh J. Burkholder K. M. (2015). *Staphylococcus aureus* strain USA300 perturbs acquisition of lysosomal enzymes and requires phagosomal acidification for survival inside macrophages. *Infect. Immun.* 84 241–253. 10.1128/IAI.00704-15 26502911 PMC4694005

[B26] Underhill D. Ozinsky A. (2002). Phagocytosis of microbes: complexity in action. *Annu. Rev. Immunol.* 20 825–852. 10.1146/annurev.immunol.20.103001.114744 11861619

[B27] Valdivia R. H. Falkow S. (1996). Bacterial genetics by flow cytometry: rapid isolation of *Salmonella typhimurium* acid-inducible promoters by differential fluorescence induction. *Mol. Microbiol.* 22 367–378. 10.1046/j.1365-2958.1996.00120.x 8930920

[B28] VanCleave T. T. Pulsifer A. R. Connor M. G. Warawa J. M. Lawrenz M. B. (2017). Impact of gentamicin concentration and exposure time on intracellular *Yersinia pestis*. *Front. Cell. Infect. Microbiol.* 7:505. 10.3389/fcimb.2017.00505 29312891 PMC5732358

[B29] Weischenfeldt J. Porse B. (2008). Bone marrow-derived macrophages (BMM): isolation and applications. *Cold Spring Harb. Protoc.* 2008:pdb.prot5080. 10.1101/pdb.prot5080 21356739

[B30] Yin C. Kim Y. Argintaru D. Heit B. (2016). Rab17 mediates differential antigen sorting following efferocytosis and phagocytosis. *Cell Death Dis.* 7:e2529. 10.1038/cddis.2016.431 28005073 PMC5261003

